# Fibration symmetry-breaking supports functional transitions in a brain network engaged in language

**Published:** 2024-09-04

**Authors:** Tommaso Gili, Bryant Avila, Luca Pasquini, Andrei Holodny, David Phillips, Paolo Boldi, Andrea Gabrielli, Guido Caldarelli, Manuel Zimmer, Hernán A. Makse

**Affiliations:** 1 Networks Unit, IMT Scuola Alti Studi Lucca, Piazza San Francesco 15, 55100-Lucca, Italy.; 2 Institute for Complex Systems (ISC), CNR, UoS Sapienza, Rome, 00185, Italy.; 3 Levich Institute and Physics Department, City College of New York, New York, NY 10031, USA.; 4 Neuroradiology Service, Department of Radiology, Memorial Sloan Kettering Cancer Center, New York, NY 10065, USA.; 5 Neuroradiology Unit, NESMOS Department, Sant’Andrea Hospital, La Sapienza University, Rome, 00189, Italy.; 6 Department of Neurology and Neuroscience, Weill Medical College of Cornell University, New York, NY, 10021, USA.; 7 Department of Radiology, Weill Medical College of Cornell University, New York, NY 10065, USA.; 8 Division of Mathematics, Computer and Information Systems, Office of Naval Research, Arlington, VA 22217, USA.; 9 Department of Mechanical Engineering, University of New Mexico, Albuquerque, NM 87131, USA.; 10 Department of Computer Science, University of Milan, Milano, Italy.; 11 ‘Enrico Fermi’ Research Center (CREF), Via Panisperna 89A, 00184 - Rome, Italy.; 12 Dipartimento di Ingegneria Civile, Informatica e delle Tecnologie Aeronautiche, Università degli Studi ‘Roma Tre’, Via Vito Volterra 62, 00146 - Rome, Italy.; 13 Department of Molecular Science and Nanosystems and ECLT, Ca Foscari University of Venice, Venice, 30123, Italy.; 14 London Institute for Mathematical Sciences, Royal Institution, 21 Albemarle St London W1S 4BS, UK.; 15 Research Institute of Molecular Pathology (IMP), Vienna Biocenter (VBC), Campus-Vienna-Biocenter 1, 1030 Vienna, Austria.

## Abstract

In his book ‘A Beautiful Question’, physicist Frank Wilczek argues that symmetry is ‘nature’s deep design,’ governing the behavior of the universe, from the smallest particles to the largest structures. While symmetry is a cornerstone of physics, it has not yet been found widespread applicability to describe biological systems, particularly the human brain. In this context, we study the human brain network engaged in language and explore the relationship between the structural connectivity (connectome or structural network) and the emergent synchronization of the mesoscopic regions of interest (functional network). We explain this relationship through a different kind of symmetry than physical symmetry, derived from the categorical notion of Grothendieck fibrations. This introduces a new understanding of the human brain by proposing a local symmetry theory of the connectome, which accounts for how the structure of the brain’s network determines its coherent activity. Among the allowed patterns of structural connectivity, synchronization elicits different symmetry subsets according to the functional engagement of the brain. We show that the resting state is a particular realization of the cerebral synchronization pattern characterized by a fibration symmetry that is broken in the transition from rest to language. Our findings suggest that the brain’s network symmetry at the local level determines its coherent function, and we can understand this relationship from theoretical principles.

## 1 Introduction

The network of internal connections crucially shapes collective phenomena in complex dynamical systems ^1^. In particular, synchronization, which is a collective behavior in which the dynamics of the network nodes converge on the same time evolution, can be exhibited either as a global state ^2^ in which all units follow the same trajectory, or via clustered states where the system splits into subsets of units synchronized to each other ^3,4^. In the latter phenomenon, known as cluster synchronization (CS) ^3,4^, a key role in determining the composition of the clusters is played by the symmetries inherent to the network structure of connections ^3–12^. This means that the symmetries of a network can theoretically predict the existence of CS emerging from a dynamics defined on the network.

Here, we find that the cluster synchronization observed in the human brain at the mesoscopic scales of regions of interest (ROIs) measured by functional magnetic resonance imaging (fMRI) is deeply intertwined with the symmetries of the brain network. These symmetries explain how the structural connections among the system’s ROIs (connectome or structural network) determine the emergent dynamical synchronization expressed in the functional network in the resting state (RS) and during a cognitive task of language.

Relating the ‘structure’ to ‘function’ is a long-standing problem in systems science ^13–17^. Earlier empirical studies of the structure-function relationship in the human brain have used diffusion tractography and fMRI to correlate white matter tracts to the functional coupling between the ROIs ^16,18–20^. Statistical analyses have shown correlations between the structural connectivity (obtained from DTI) and resting state functional connectivity (obtained from fMRI) between anatomically defined ROIs ^16,18–20^. While the structural connectivity partially resembles the resting state functional connectivity; two ROIs can be structurally connected but not functionally related, and vice versa.

More recently, the structure-function relation has been investigated by neurodynamical modeling of fMRI signals in resting and task-based cognitive states ^21,22^. These models are validated by comparing predicted spatiotemporal patterns with empirical functional connectivity data. Geometric constraints of curvature and distance have been shown to shape both the spontaneous and induced activity of the brain ^23^. These latest results suggest a principled theoretical approach to understanding how structure shapes function is possible.

In this paper, we postulate that a symmetry theory of the connectome sheds light on how structure determines function by predicting the synchronization of the brain ROIs. We show that the theory of symmetry— and symmetry-breaking ^24^— widely used in physics ^25–27^, geometry ^28^, dynamical systems ^3–6^, and geometric deep learning ^29^, can bridge the gap between the brain network structure and its dynamic synchronization.

The symmetries we find in the human brain are not those of physical systems. Physical (and geometrical) symmetries are automorphisms and form symmetry groups ^25,26^. These are global symmetries since they preserve the global shape of objects, and, in the particular case of graphs, they are permutations of nodes that preserve the global adjacency of nodes. Instead, the symmetries we find in the brain network are symmetry fibrations ^7,11^— derived from Grothendieck fibrations in category theory ^30^— which form symmetry groupoids ^5,6^. Fibrations are less restricted symmetries than automorphisms because they are local symmetries that preserve only the color-isomorphic inputs of nodes. Consequently, they preserve the dynamical evolution leading to cluster synchronization in the network.

Fibrations have been proven to be a useful tool for describing how genetic networks are built from the bottom up to process information through gene expression ^5,10–12,31^. They also appear in simple *C. elegans* neural circuits ^10,32–34^, and are crucial in explaining the expressiveness and power of graph neural networks ^35,36^.

Here, we expand this view to the human brain, letting the empirical activity of synchronization drive the inference of the underlying connectome. We implement a symmetry-driven algorithm based on a mixed integer linear programming to infer the structural network that sustains the cluster synchronization of the functional language network (a brain’s specific cortical sub-network involved in the language function ^17^) obtained experimentally in different processes whose outcome is the human language ability.

In analogy to the theory of phase transitions between states in physical systems ^24,37^, we describe the recruiting of communication resources across different brain states as a process of network-symmetry breaking. First, we find that the baseline connectome of the language network displays a global group symmetry that switches to a local fibration symmetry to sustain the resting state synchronization dynamics. Then, this symmetry is further broken by the activity-driven lateralization induced by the language task. The brain switches from the resting state to the execution of language inducing a fibration symmetry breaking of the connectivity pattern sustaining the synchronization of the brain regions.

## 2 Cluster synchronization in the functional network

### 2.1 Functional network of synchrony between ROIs from fMRI.

**Figure 1:**
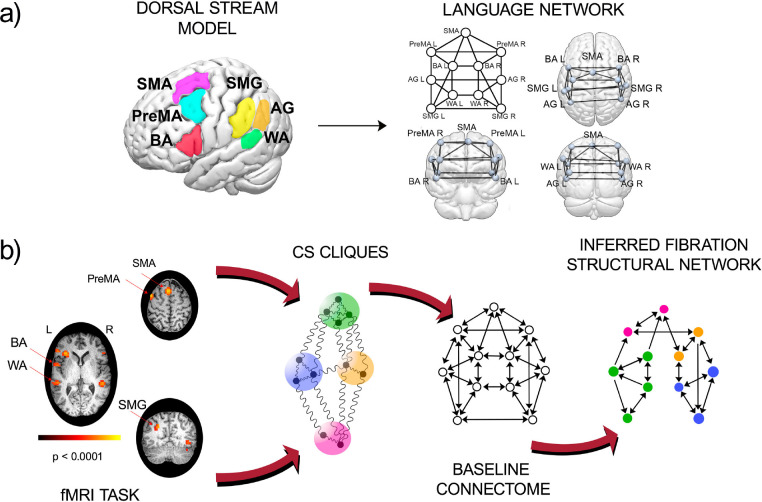
Dual stream model of language and the inference scheme. **(a)** Left: ROIs of the primary language network is given by the dorsal stream of the dual-stream model localized in the 3d brain. Right: dual (dorsal) stream baseline connectome showing the fiber tracks between the ROIs in **(a)**. **(b)** Pipeline for inference of the structural network from CS data. Left: fMRI images for RS or a task over many subjects are taken as input to calculate the group-average CS cliques among ROIs. The CS are identified with the colors in the baseline connectome. A mixed integer programming algorithm is employed to optimally infer the structural network (right) that sustains the coloring cluster pattern obtained from the dynamics.

We analyze fMRI BOLD signals from n(=20) subjects (normal, healthy volunteers with no neurological or psychiatric morbidities) at rest and while performing two language tasks to construct functional networks associated with expressive language. We build functional networks as a group average over subjects performing two well-studied language tasks, phonemic fluency, and verb generation ^38^, and at rest ^39^ (Fig. 1 and Methods Sec. 6.3). During the phonemic fluency task, the subjects are asked to silently generate as many words as possible, starting with a given letter. During the verb generation task, subjects are asked to generate action words associated with the presented nouns. During the resting state, subjects are instructed to lie in the scanner with their eyes open, try to think of nothing in particular, and fixate on a central cross on a screen.

The functional network is built between anatomically defined ROIs that are primarily involved in language according to the dual stream model ^40,41^ (see Fig. 1a and Methods Sec. 7 for more details). Specifically, we consider the dorsal stream of the dual stream model in our analysis: Supplementary Motor Area (SMA), Premotor Area (PreMA, left and right), Supramarginal Gyrus (SMG left and right), Broca’s Area (BA, left and right), Angular Gyrus (AG, left and right), and Wernicke’s Area (WA, left and right). Many other secondary areas of the brain are involved in language. This gets more complicated in pathological states such as brain tumors that lead to language reorganization. Here, we focus our analysis on these primary language areas and their connections.

We use standard methods to build the functional network from the time-dependent fMRI-measured blood-oxygen-level-dependent (BOLD) signal ^15^ (see Methods Sec. 8). For a single subject, we measure synchronization using the Phase-Locking Value (PLV) ^42^ among the BOLD time series between ROI pairs (see Extended Data Fig. 5 and Methods Sec. 9). We obtain the correlation matrix observed in Extended Data Fig. 6 for a typical subject. Averaging these matrices across n subjects, we obtain a weighted group-average correlation matrix with edge weights in the [0,1] range. Using this correlation matrix, we obtain the functional network from which the CS of ROIs are obtained.

Ideally, a perfect CS is a non-overlapping, fully connected induced subgraph (clique) embedded in the functional network. Since this ideal synchronization cannot be expected from real data; we relax this condition by allowing the fully connected subgraph to be connected by weak interclique links. We define a CS N-clique as the induced, fully connected subgraph of the functional network composed of N nodes that satisfy the following conditions:

(1) $$\begin{array}{l} \sum_{i<j}^{1,N}\sigma \left(x_{i}\left(t\right),x_{j}\left(t\right)\right)\geq \frac{N\left(N\;-\;1\right)}{2}\sigma \left(x_{k}\left(t\right),\;x_{k^{\prime }}\left(t\right)\right)\\ \;\forall k=1,\ldots ,N\;\mathrm{and}\;k^{\prime }\in {ℳ}_{k}, \end{array}$$

where ${ℳ}_{k}$ is the set of nearest neighbors of node k=1,…,N not belonging to the considered clique, and $\sigma \left(x_{i}(t),x_{j}(t)\right)$ is the PLV of the functional time series $x_{i}(t)$ and $x_{j}(t)$ of nodes i and j, respectively (see Extended Data Fig. 5 and Methods Sec. 9 for further details).

The clusters of synchronized ROIs are obtained by applying a standard percolation threshold procedure ^43,44^ to the correlation matrix. Starting from a disconnected graph, links between nodes are progressively added in decreasing order of weight of the correlation matrix (i.e., synchronization), starting from the largest one. A CS clique is found as soon as the condition in Eq. (1) is satisfied. The process stops when the weight of the links to add doesn’t allow further cliques to form. This process defines a hierarchy of CS according to the order of clique appearance in the percolation process.

### 2.2 Cluster synchronization in resting state and task.

**Figure 2:**
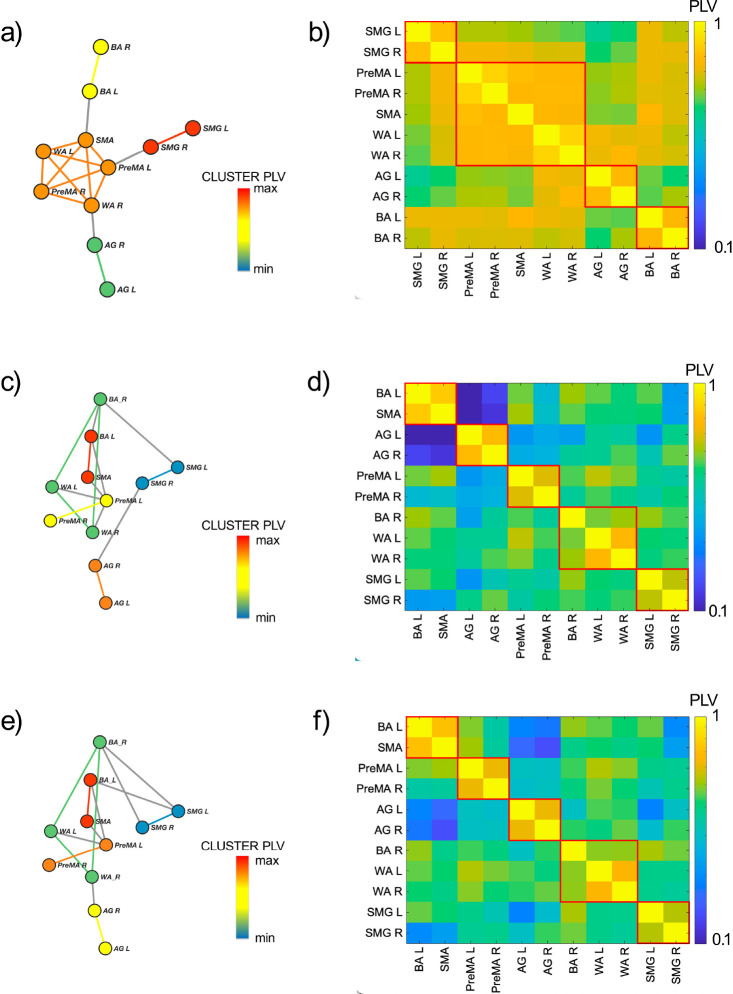
Functional networks and cluster synchronization. **(a)** Functional network during resting state shows the CS in different colors. **(b)** Phase Locking Value matrix for the eleven ROIs during resting state. **(c)** Functional network during phonemic fluency language. **(d)** Phase Locking Value matrix during phonemic fluency task. **(e)** Functional network during the verb generation language task. **(f)** Phase Locking Value matrix during verb generation task. In the three networks **(a)**, **(c)** and **(d)** nodes and edges are colored according to the Cluster PLV color bar reported aside. Cluster PLV is calculated as the average PLV over links for each CS found in the network. Grey edges connect clusters. The red-lined boxes in **(b)**, **(d)** and **(e)** are visual indicators for the CS and clusters are shown in decreasing order of Cluster PLV.

The RS-CS is calculated from the functional network between the ROIs defined in Sec. 2.1 and the correlation matrix built from their fMRI signals obtained in the RS experiments (see Methods Sec. 10). The result for the group average correlation matrix is shown in Fig. 2b. Using this correlation matrix, we obtain the functional network displayed in Fig. 2a with the synchronized clusters of ROIs from Eq. (1) is shown in different colors. It is known that the RS functional network is approximately left-right symmetric ^45,46^. Our results confirm this evidence by demonstrating bilateral synchronization of three CS (Fig. 2a). Each comprises a bilateral pair of regions (supramarginal gyrus, angular gyrus, and Broca). Beyond this expected result, we find a novel central CS composed of a pentagonal clique of two bilateral pairs of regions (premotor and Wernicke’s area) and the supplementary motor area. This CS fits the auditory-motor integration mechanism of the dorsal stream of the language processing model (see Methods Sec. 10 for more details).

A different functional network is activated when the subject performs a language task. We find that a common feature of both verb generation (Fig. 2 c and d) and phonemic fluency (Fig. 2 e and f) networks is the emergence of left lateralization ^17^ by the engagement of BR left and left frontal language areas in the task. We find that the BA left area is recruited by SMA, becoming, in the process, desynchronized with BA right, which, in turn, synchronizes with WA left and right. The CS set is identical in both language tasks (Figs. 2 c and e), yet the clusters appear in different orders in the CS hierarchy. During verb generation, the bilateral PreMA cluster is more synchronized than the AG one, while things are reversed during the phonemic fluency task. Finally, the two less synchronized clusters are the same in both tasks: the triangle composed of the bilateral WA, the right BA, and the bilateral SMG. These results are consistent with the current understanding of language modeling (see Methods Sec. 10).

## 3 Theory of global and local symmetries

### 3.1 Automorphisms and fibration symmetries.

**Figure 3:**
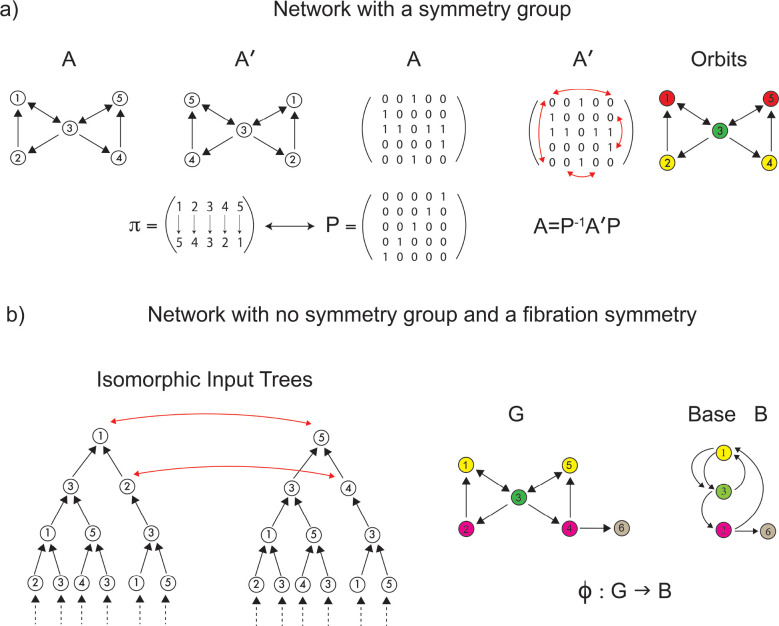
Symmetry Formalism. **(a)** Example of automorphism in a graph with group symmetry. Left: A permutation π transforms the graph A into $A^{\prime }$. This can be written down in matrix notation through a permutation matrix P. The permutation is a symmetry when $A=A^{\prime }$. Right: applying all symmetries to every node generates the orbital partition shown in the colored nodes. **(b)** Example of fibration symmetry in a graph with no group symmetry. The addition of the outgoing edge from node 4 to 6 in **(b)** destroys the global automorphism in **(a)**. Yet, the symmetry fibration still remains since there are nodes (nodes 1 and 5 and nodes 2 and 4) with isomorphic input trees (left). The fiber partition is shown in graph G: *(i)* nodes with the same colors are in fibers, *(ii)* are balanced because they receive the same colors from neighbors, and *(iii)* are synchronized under any dynamics. The fibration ϕ collapses the fibers into the base B by following the lifting property (right).

Once we have specified the pattern of CS within the language functional network, we present a symmetry theory to infer the structural language network that sustains the observed synchronization. Classically, symmetries are mathematically captured by **automorphisms** ^47^. In a graph, an automorphism is a permutation of the nodes of the graph that preserves the global adjacency connectivity (Fig. 3a and Methods Sec. 11). That is, the (in-coming and out-going) neighbors of *every* node are preserved by the permutation: note that this is a *global* condition because the map involves all nodes. The clusters of nodes subjected to these permutations are called **orbits**, and nodes within each orbit synchronize their activity under a dynamical system admissible for the network. The requirements for the existence of orbits are hard (i.e., difficult to satisfy) and **global**, as automorphisms must preserve the entire adjacency matrix.

Conversely, a graph homomorphism called **graph fibration** ^7,11^, allows for the definition of less constrained (i.e., more general) **local** symmetries than do automorphisms (Methods Sec. 12). Graph fibrations are derived from the categorical notion with the same name, introduced by Grothendieck and others in the 1960’s ^30^.

#### Definition 1 (Fibration).

Given a graph $G=\left(N_{G},E_{G}\right)$, a graph fibration ϕ of G to a base graph $B=\left(N_{B},E_{B}\right)$ is a homomorphism (Fig. 3b, right)

(2) ϕ:G→B,

that satisfies the following *lifting property* ^7^:

#### Definition 2 (Lifting Property).

For any edge $e_{B}\in E_{B}$ and any node $n_{G}\in N_{G}$ such that $\phi \left(n_{G}\right)=t\left(e_{B}\right)$ (where t is the function that specifies the target node of each edge), there is a unique $e_{G}\in E_{G}$, called the lifting of $e_{B}$ at $n_{G}$, such that

(3) $$\phi \left(e_{G}\right)=e_{B}\;\text{and}\;t\left(e_{G}\right)=n_{G}.$$

Otherwise, a fibration is a graph homomorphism that is only required to be a bijection of *local in-neighborhoods* rather than of the entire network. Therefore, it is truly a local symmetry and much less constrained than the global symmetries of automorphisms.

An equivalent, and perhaps more intuitive, definition of graph fibration was given in ^11^ and grounds on the concept of an input tree of a graph’s node (see Fig. 3b, left).

#### Definition 3 (Input tree).

The input tree for a node v, denoted T(v), is a rooted tree centered at node v. The first layer of the tree is the node’s in-neighborhood, called its input set. Each subsequent layer is then iteratively defined as the input set of the input set.

The input tree represents the complete set of all paths that terminate on v and thus represents the dynamical history of the information flow arriving at v through the network. Accordingly, we can use the input tree to define cluster synchronization in the network.

A fibration ϕ of G “collapses” the nodes of G with isomorphic input trees into the base B, see Fig. 3b right. A fibration that produces the minimal base (i.e., maximum collapse) is referred to as the **symmetry fibration** of G because it collects all the symmetries of the network ^11^. Clustered nodes with isomorphic input trees are called **fibers** (the colored nodes in Fig. 3b) and are analogous within the fibration framework to group orbits in the automorphisms world. (Note: ‘fiber’ in the context of fibration should not be confused with ‘fiber’ in the context of ‘fiber-tracks’).

A fundamental theoretical results proved by DeVille and Lerman ^8^ [Theorem 4.3.1 and Lemma 5.1.1] (see also ^5,9^) has profound consequences for the structure-function relation by allowing the gap between the fibration of the graph (structure) and the existence of CS (function) to close:

#### Definition 4 (Fiber synchrony).

The set of nodes in the same fiber of the fibration (i.e., with isomorphic input trees) is proven to be synchronous under a dynamical system defined on the network. This result is quite general since it is independent of the type of dynamics considered, as long as it is admissible with the graph.

The partition of nodes into fibers of a fibration coincides with the partition obtained by **balanced coloring**, or equitable partition ^5–7^. This correspondence provides a third alternative definition of a graph fibration in terms of the input sets rather than the input trees:

#### Definition 5 (Balanced coloring = fiber synchrony = CS).

*A* balanced coloring *of a graph is an assignment of colors to nodes, such that nodes of one color receive the same amount of the other colors from their in-neighbors (i.e., same number of in-neighbors of each other color, see Fig. 3b, right)*.

Aldis ^48^ [Theorem 4.2 and Corollary 4.3] has indeed shown that the fibers of the fibration are the partition induced by balanced colorings of the graph. Thus, we identify the CS obtained from the dynamics with the fibers of the graph or analogously the balanced coloring.

The orbital partition obtained from automorphisms (Fig. 3a, right) is also a balanced coloring but is generally finer than the coarsest balanced coloring determined by the symmetry fibration: i.e., every orbit is a fiber, but not every fiber is an orbit. This implies that a graph may have more fibration symmetries than those induced by the automorphisms (Fig. 3b).

In other words, all automorphisms are fibrations, but not all are automorphisms. Intuitively, the conditions imposed by automorphisms, being on non-local scales, are much harder to satisfy than in vibrations, which preserve only the local in-neighborhood. Algorithms to efficiently calculate the minimal balanced colorings (fibers) in a network exist ^11,12,49^. They are also widely used in machine learning and GNN as the Weisfeiler-Lehman graph isomorphism test ^35^. Orbits and automorphisms are calculated with McKay’s Nauty algorithm ^50^.

### 3.2 The symmetries of the dual stream baseline connectome of language.

Having defined symmetries, we now look for them in the connectome of white-mater fiber tracks between the ROIs primarily involved in language ^51^. The known bundles of axonal tracks between ROIs in the dorsal stream model is shown in the connectome displayed in Fig. 1a, right (see Methods Sec. 13 for more details). They are those of the dorsal stream formed by white-matter tracks of the superior longitudinal fasciculus (SLF) arcuate fasciculus (AF) system. In brief, the AF connects the inferior frontal gyrus (Broca’s area) to the posterior superior temporal gyrus (Wernicke’s area). The SLF connects Broca’s area and premotor area to the inferior parietal areas (supramarginal and angular gyri) ^51,52^. The frontal aslant tract (FAT) connects Broca’s area with the supplementary motor area, serving the verbal fluency components of language ^53,54^. Sensorimotor integration culminates in the Broca’s area and ventral PreMA, which are responsible for articulatory planning ^55,56^. Two parallel dorsal pathways ^17^ connects the PreMA (dorsal pathway I) and Broca (dorsal pathway II) to Wernicke in order to predominantly support sound-to-motor mapping. The second supports higher-level language processes.

**Figure 4:**
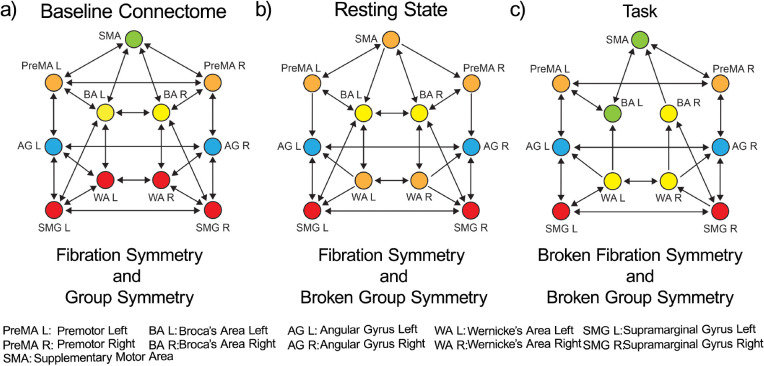
Breaking of symmetry from the baseline connectome to inferred RS network to task network. **(a)** Minimal balanced coloring in the baseline connectome. This network has the highest symmetry: a global automorphism group, which is the same as the local fibration symmetry with five orbits equal to fibers (five balanced colors). **(b)** Inferred RS structural network using the CS from Fig. 2a. The network has only local fibration symmetry with four fibers but no global symmetry, which is broken with respect to the connectome in **(a)** under the RS dynamics. **(c)** Inferred language task network from the coloring in Fig. 2c or e (which are the same). The lateralization of function under the language task breaks the fibration symmetry of **(b)** showing less symmetry (more fibers than RS). The group symmetry remains broken.

These tracks constitute the *dual stream (dorsal) baseline connectome* of language shown in Fig. 1a. A symmetry analysis of this connectome reveals a remarkable symmetry (Fig. 4a): in such a network, the number of fibers and orbits are equal (equal to five; see Methods Sec. 14). This means that the automorphisms (symmetry group) and the fibration symmetries of this network are the same, implying that the global symmetry is the same as the local.

According to both orbital and fiber partitions, the five fibers (orbits) are (Extended Data fig. 7): a 4-ROI cluster composed of WA (left and right) and SMG (left and right), and fibers respecting the left-right symmetry: PreMA (left and right), BA (left and right), AG (left and right), and SMA (alone).

For instance, the input trees of ${WA}_{L}$, ${WA}_{R}$, ${SMG}_{L}$ and ${SMG}_{R}$ are isomorphic (Extended Data Fig. 7c). Therefore, these ROIs belong to the same fiber and are collapsed by fibration symmetry. At the same time, the permutation that maps ${WA}_{L}$ to ${SMG}_{L}$, and ${WA}_{R}$ to ${SMG}_{R}$ (displayed in Extended Data Fig. 7b) in cycle notation: $\pi_{2}=\left({WA}_{L}{SMG}_{L}\right)\left({WA}_{R}{SMG}_{R}\right)$ is also an automorphism marking the presence of the global permutational symmetry. This creates the fiber (= orbit) colored red in Fig. 4a. This fibration=automorphism situation is a condition of high symmetry. It means an intrinsically highly symmetric network represents the highway of inter-regional communicability that allows language processing to emerge.

Given this initial baseline symmetric connectome, a stable pattern of synchronization can emerge during a functional engagement (Fig. 2) that should induce a modification of the symmetries needed to sustain each functional synchronization. Hence, breaking this high initial symmetry is expected to be a crucial condition for effective functional activity. Lower symmetric states are expected when the orbits are more than the fiber (indicating a loss of global group symmetry) or when the number of fibers increases, indicating a loss (breaking) of local fibration symmetry. We explore these cases next.

### 3.3 Inferring the structural network sustaining RS and language from cluster synchronization.

The baseline connectome represents the set of available routes composing the primary information highway of the brain involved in language. However, which routes of this highway are utilized depends on the type of task to which the brain responds ^13^. The main hypothesis postulated in ^13^ is that the brain’s functional activity utilizes a subset of the links available in the ‘highway’ connectome to operate in each functional state. This ‘one-to-many’ degenerate structure-function relation ^13^ allows the emergence of diverse functional states (resting, language, etc.) from a unique static connectome architecture. In the present case, it means that, given the dorsal stream baseline connectome in Fig. 1a, different subsets of this connectome mediate different functional networks ^13,14^. We demonstrate this structure-function relation by matching the patterns of ROI synchronization and coloring clustering obtained from Fig. 2 to different realizations of the structural network.

Accordingly, we infer the structural network associated with each balanced coloring of the functional network obtained experimentally in RS and task. To this end, we develop a mixed integer linear programming (MILP) ^33,57^ algorithm to optimize a minimal link removal from the connectome to satisfy the balanced coloring obtained in the experiments. The ‘one-to-many’ hypothesis is falsifiable. If true, MILP must find a solution to the color partitioning using only removals. If there is no solution, then the hypothesis is wrong.

The inference algorithm can be summarized in the following steps (Fig. 1 and Methods Sec. 15):

- For a given set of ROIs (Fig. 1a left), identify the baseline connectome that form the graph of all permitted structural connections among them (Fig. 1a right);
- Using the PLV synchronization measure, find the CS from the functional network according to Eq. (1) for a given task (Fig. 2a, c, e). Assign to each ROI in each CS in the functional network a color symbolizing the fiber partition or balanced coloring.
- Decimate the baseline connectome by removing the minimal number of edges until the fibers of the decimated graph match the coloring obtained from the functional network (Fig. 1b right).

We apply this algorithm to identify the routes that sustain the functional network at rest and during the execution of the two language tasks. Although the ranking of the CS is different for the two tasks, the coloring is not. It means the structural network that sustains the two types of functional activity in language is the same.

## 4 Symmetry-breaking transition to resting state and task

While symmetry principles stand as crucial elements within natural laws, much of the world’s complexity emerges from mechanisms of symmetry breaking, which encompasses various ways nature’s symmetry can be veiled or disrupted ^24,37^ (Methods Sec. 16). Any situation in physics in which the ground state (i.e., the state of minimum energy) of a system has less symmetry than the system itself, exhibits the phenomenon of spontaneous symmetry-breaking. For instance, different phases of matter are characterized by different symmetries. At higher temperatures, matter takes on a ‘higher symmetry’ phase (e.g., paramagnetism, normal conductivity, and fluidity), while at lower temperatures, the symmetries of the phases are broken to ‘lower symmetry’ (e.g., ferromagnetism, superconductivity, and superfluidity).

Although the connectome is not a dynamic state per se, we can explain the transitions from the baseline highway of connections to its subset responsible for sustaining the communication processes at rest and task analogous to symmetry breaking in ferromagnets. Starting from the baseline connectome with high symmetry configuration as estimated by orbits and fibers (Fig. 4a), we find progressive and different symmetry-breaking processes in the structural connectivity as the brain engages in different states (Fig. 4b and c).

The first symmetry-breaking transition occurs once the dynamics are introduced. Figure 4b shows the balanced coloring of the inferred structural network sustaining the resting state synchronization. A symmetry analysis of this network (see Methods Sec. 17 and Extended Data Fig. 8) shows that while in the baseline connectome, we have both fibrations and automorphisms, in the resting state condition, the group symmetry, including the global left-right symmetry, is lost, and the fibration symmetry is enhanced. We find four fibers in the resting state (four colors in Fig. 4b) vs. five fibers found in the baseline connectome (Fig. 4a).

When synchronization processes intervene, the symmetry is broken in the precise direction of the optimal communicability among the brain regions. The resting state dynamics introduce a mismatch between orbits and fibers. Fibration symmetry increases (fewer fibers) during the resting state synchronization (Extended Data Fig. 8a left and 8c), while a total loss of group symmetry is produced (Extended Data Fig. 8a right and 8b). Remarkably, while the global left-right symmetry is disrupted in the RS connectome, the local left-right fibration symmetry necessary for left-right synchronization is still maintained. This suggests that the perturbation represented by brain synchronization on the static network neutralizes the automorphism, but reinforces the biological fibration configuration, which in turn allows the stability of the synchronized dynamics.

Figure 4c shows the balanced coloring of the inferred structural network engaged in the language (see symmetry analysis in Methods Sec. 18 and Extended Data Fig. 9). During the execution of the task, the activity is largely polarized in recruiting areas devoted to the correct functioning. The lateralization of brain activity during language execution induces a further fibration symmetry-breaking between the Broca left and Broca right areas, which now belong to two different fibers as seen in Fig. 4c. Broca left is recruited by the SMA, while Broca right is recruited by the Wernicke pairs, which remain locally symmetric. The number of fibers is increased to five (less symmetry) compared to the fibration symmetry in RS, as if the activity induced by the task execution acts as a perturbation over the resting state dynamics. The global symmetry remains completely broken, presenting only the trivial (identity) automorphism, and one orbit per ROI (Extended Data Fig. 9a right and 9b).

The five fibers found and the lateralization characterizing them are compatible with the neurocognitive models of the functional circuits of language production. Indeed, studies have demonstrated that networks involving the temporal cortex and the inferior frontal cortex, predominantly lateralized to the left hemisphere, are implicated in supporting syntactic processes, while temporo-frontal networks with less lateralization are involved in semantic processes ^58,59^. Thus, the symmetry-breaking is found to be a direct consequence of cognitive specialization of brain areas (specifically the group SMA, BA, and WA), for the elaboration of specific tasks (i.e., syntactic tasks) as it happens also to other regions of the brain that give place to a recognized brain asymmetry ^60^.

The description of the brain region’s recruitment during a task execution as a symmetry-breaking process is only possible because the pattern of connections that support the communication among such regions change selectively according to the specific conditions in which the brain is. Different dynamics can be matched with different patterns of structural connectivity unveiled by symmetry considerations. As a consequence, the mesoscopic matching of the brain’s structural-to-functional connectivity emerges as a reconfiguration process driven by the fibration symmetry induced by the communication dynamics among brain regions.

## 5 Discussion

We propose a symmetry theory of brain connectivity whose possible functional transitions can be pooled in determined sets of breaking symmetry processes. The primary application of the synchronization-driven inference method proposed here is the understanding of disease pathways. The inference of pathways from dynamical data on healthy subjects can be extended to neurological or psychiatric conditions, allowing the identification of differential disease pathways, leading to an understanding of the disease, establishing the diagnosis, and ameliorating the consequences. Moreover, our method can be beneficial for drug development by targeting the inferred structural network of a specific disease onto a healthy one. Finally, the controllability of brain networks, which is an open problem in neuroscience should find a boost from the results reported here. The treatment of neurological and psychiatric diseases through invasive (surgery) or non-invasive (electric/magnetic stimulation) intervention ^38^ will benefit from the identification of the patterns of symmetry and synchronization and their breaking processes to reduce side effects or to optimize the effectiveness of the application.

Overall, our findings suggest that the brain’s local symmetry at the mesoscopic level determines its coherent function. Symmetry fibrations strictly generalize the symmetry groups of physics and have been found in biological systems from the human brain and *C. elegans* connectome to genetic and metabolic networks. Thus, if symmetry fibrations can be postulated to be ‘nature’s deep design’, they will unify not only physics but also biology, providing a plausible solution to the aforementioned conundrum.

## Methods

### 6 Experimental protocols

#### 6.1 Subjects.

Twenty healthy right-handed subjects (mean age= 37, SD=12; 7 females and 13 males) without any neurological history participated in the study. The study was approved by the Institutional Review Board at Memorial Sloan Kettering Cancer Center, in compliance with the declaration of Helsinki and informed consent was obtained from each subject.

#### 6.2 MRI methods.

A GE 3T scanner (General Electric, Milwaukee, Wisconsin, USA) and a standard quadrature head coil was employed to acquire the MR images. Functional images covering the whole brain were acquired using a (T2*)-weighted imaging sequence sensitive to blood oxygen level-dependent (BOLD) signal (repetition time, TR/TE = 2500/40 ms; slice thickness = 4.5 mm; matrix = 128 × 128; FOV = 240 mm; volumes = 160). Functional matching axial T1-weighted images (TR/TE = 600/8 ms; slice thickness = 4.5 mm) were acquired for anatomical co-registration purposes.

#### 6.3 Language tasks and RS.

All subjects performed a resting-state task, a verbal fluency task using verb generation in response to auditory nouns and a phonemic fluency letter task in response to task instructions delivered visually.

During the resting state condition, subjects are asked to lie in the scanner and to keep their eyes open, to try to think of nothing in particular, and to keep fixating on a central cross on a screen during the RS.

In the verb generation task, subjects were presented with a noun by oral instruction and then asked to generate verbs associated with the noun. For example, subjects are presented with a noun (e.g., ‘baby’) and asked to generate verbs (e.g., ‘cry,’ ‘crawl’) associated with the noun. Subjects perform the task silently to avoid motion artifacts. Four nouns are displayed over eight stimulation epochs, each lasting 50 s, allowing 32 distinct nouns to be read over the entire duration. Each epoch consisted of a resting period (30 s) and a task period (20 s).

In the phonemic fluency task, on the other hand, subjects are asked to generate nouns that begin with a given letter silently. For instance, the subject presented with the letter ‘A’ may generate words such as ‘apple,’ ‘apron,’ or ‘ashtray.’ Stimuli are displayed on a screen over eight stimulation epochs, each lasting 20 s. During the task, two letters are presented in each stimulation epoch. Each epoch also consisted of a 30-second resting period during which subjects were asked to focus on a blinking crosshair.

In order to avoid artifacts from jaw movements, subjects were asked to silently generate the words.

#### 6.4 Data preprocessing.

Functional MRI data were processed and analyzed using the software program Analysis of Functional NeuroImages (AFNI; Cox, 1996). Head motion correction was performed using 3D rigid-body registration. The first volume was selected to register all other volumes. The first volume was chosen because it was acquired before the anatomical scan. During the registration, the motion profile was saved and regressed. Spatial smoothing was applied to improve the signal-to-noise ratio using a Gaussian filter with a 4 mm full width of half maximum. Corrections for linear trend and high-frequency noise were also applied. Resting-state data requested some more preprocessing steps. They were corrected for head motion by regressing head motion data and the first five principal components of the white matter and CSF signals. They were also detrended, demeaned, and band-pass filtered (frequency range 0.01–0.1 Hz). All fMRI data were registered to the standard space (Montreal Neurological Institute MNI152 standard map). Task data for task state synchronization analyses were additionally preprocessed using a general linear model. The stimulation scheme was removed by fitting the task timing (block design) for each condition. This was accomplished using the convolution of the block design with a standard 2-gamma hemodynamic response function used for the task activation estimates, fit simultaneously with its derivative.

### 7 Definition of ROIs: dorsal stream model of language

The modeling of language processing has been based for a long time on the Geschwind-Lichteim-Wernicke model ^61^, primarily drawn from observations of individuals with brain injuries. Following this model, words are perceived through a dedicated word reception center (Wernicke’s area) within the left temporoparietal junction. Subsequently, this region sends signals to a word production center (Broca’s area) in the left inferior frontal gyrus.

Advancements in electrophysiological and MRI techniques have unveiled a dual auditory pathway. This led to the development of a dual stream model ^40,41^. According to this model, two distinct pathways connect the auditory cortex to the frontal lobe, each serving different linguistic functions. The auditory ventral stream pathway is responsible for sound recognition and is called the auditory ‘what’ pathway. On the other hand, the auditory dorsal stream, found in humans and non-human primates, is responsible for sound localization and is called the auditory ‘where’ pathway. In humans, particularly in the left hemisphere, this pathway also handles speech production, repetition, lip-reading, phonological working memory, and long-term memory.

The relevant ROIs are those areas involved in the two language tasks considered. Since the tasks are both focused on language production (phonemic fluency and verb generation), regions of the dorsal stream are part of the analysis (Fig. 1): Supplementary Motor Area (SMA), Premotor Area (PreMA, left and right), Supramarginal Gyrus (SMG left and right), Broca’s Area (BA, left and right), Angular Gyrus (AG, left and right), Wernicke’s Area (WA, left and right). The BA and WA are recognized as responsible for language expression and comprehension. The supplementary motor area (SMA) has been largely considered involved in controlling speech-motor functions, and it has also been shown ^62^ that the SMA performs several higher-level control tasks during speech communication and language comprehension. The AG is assumed to be a region of the brain associated with complex language functions (i.e., reading, writing, and interpretation of what is written). In contrast, the SMG is involved in the phonological processing of high-cognitive tasks. Finally, processing an action verb depends in part on activity in a motor region that contributes to planning and executing the action named by the verb. The premotor cortex is known to be functionally involved in understanding action language ^63^.

### 8 Structural and functional network

We distinguish between the ‘structural network’ and ‘functional networks’ of the brain ^13,15,16^. The ‘structural network,’ also called ‘connectome’ or ‘structural graph’, is a set of nodes and edges that form the brain’s underlying network of physical connections. We study the brain graph at mesoscopic scales where nodes are ROIs defined at the mm scale (measured by fMRI in the human brain) and edges are the white matter tracks that connect the ROIs. These edges are usually measured by diffusion tensor imaging (DTI) or are known from the literature. By structure, we mean the structure of this graph.

When a graph is equipped with state variables and dynamical equations, it technically becomes a ‘network system’ of ODEs. Specific features of the dynamics, such as the quantitative value of interaction constants or the frequency of an oscillation, depend on the precise details of the model equations. Here, we focus on more general features, which can occur for broad classes of models and systems. Synchronization is the prime example of such a feature. We associate ‘function’ with the synchronization of ROIs measured from fMRI indicating that the ROIs are functionally related. This synchronization occurs in clusters of ROIs or CS ^3–6^. The ‘functional network’ from where CS is obtained is built from the synchronization between ROI activity in the brain as measured by fMRI. ^13,14^.

### 9 Cluster synchronization and the functional network

BOLD time series were extracted from all voxels in a sphere of radius 6 mm centered on target MNI152 coordinates addressing a ROI. Each ROI was composed of 123 voxels. The synchronization between pairs of nodes of the language network was estimated as the Phase Locking Value (PLV) ^64^ between the BOLD time series from pairs of ROIs. Once time series were obtained for the eleven ROIs included in the study (by spatial averaging the BOLD signal within each ROI at each time point), the synchronization was calculated as follows. Given the BOLD signals $n_{u}(t)$ and $n_{v}(t)$ coming from regions u,v=1,…,N(N=11), their instantaneous phases $\phi_{n_{u}}(t)$ and $\phi_{n_{v}}(t)$ can be obtained by means of their Hilbert transform (see Extended Data Fig. 5). The PLV $\sigma (\phi_{n_{u}}(t),\phi_{n_{v}}(t))$ is then given by:

(4) $$\sigma \left(\phi_{n_{u}}(t),\phi_{n_{v}}(t)\right)=|{\left\langle e^{-j\left(\phi_{n_{u}}(t)-\phi_{n_{v}}(t)\right)}\right\rangle }_{t}|,$$

where j is the imaginary unit.

To test the statistical significance of the PLVs, a non-parametric permutation test was run by generating surrogate ROI signals randomly re-arranged and eventually time-reversed (1,000 permutations). This procedure allowed the generation of a null distribution that shared the same parameters (mean and standard deviation) of the original data and similar (but not identical) temporal dynamics. This produced a null distribution of t-statistics that provided the one-tailed P value. P values were estimated using a generalized Pareto distribution to the tail of the permutation distribution ^65^. Correction for multiple comparisons was provided by thresholding statistical maps at the 95th percentile (P<0.05, FDR) of the maximum t distribution from the permutation ^66^.

The PLVs were then entered in a N×N correlation matrix, representing the correlation/synchronization or PLV matrix. Finally, the PLV matrices were averaged across subjects in each experimental condition (resting state, phonemic fluency task, verb generation task). The functional network is then obtained by thresholding the group-averaged correlation matrix, obtaining the CS as explained in the main text.

The Cluster PLV shown in Figs. 2a, c, and e is the value of the weight of each link within a CS, and it is calculated as the average PLV across the edges composing the CS clique. This Cluster PLV represents the strength of the synchronization within each CS and defines the hierarchy of CS according to its strength.

### 10 Cluster synchronization in resting state and tasks

The patterns of CS found within the language network allow discriminating the resting state condition from the task ones. RS-fMRI demonstrates sub-optimal characterization of both language dominance and lateralization of eloquent areas ^45^, due to enhance homotopic synchronization. This is especially true in networks with left-right symmetry, such as those involved in motor and vision, as well as in language, which is normally lateralized (breaking the left-right symmetry) during the execution of the task ^46^. Our results confirm this evidence by demonstrating high left-right symmetry of the language network during resting-state. We find, according to a descendent synchronization hierarchy, a CS composed of bilateral SMG, a CS composed of bilateral BA, a pentagonal CS composed of two bilateral pairs of regions (PreMA and WA) and the SMA, and a CS composed of bilateral AG (Fig. 2 a).

The clusters that we find in RS are hierarchically ordered according to the Cluster PLVs as follows (Fig. 2 a and b): the pair {SMG L, SMG R}, [PLV = 0.762], the pentagon {PreMA L, PreMA R, SMA, WA L, WA R}, [PLV = 0.712] the pair {AG L, AG R} [PLV = 0.689] and the pair {BA L, BA R}. [PLV = 0.689]. The inter-cliques connections were characterized by PLV values smaller than the Cluster PLVs: (AG R, WA R) with [PLV = 0.682], (BA L, SMA) with [PLV = 0.662] and (SMG R, PreMA L) with [PLV = 0.639].

The large pentagonal synchronization clique composed of SMA, PreMA (bilateral), and WA (bilateral) fits the auditory-motor integration mechanism of the dorsal stream of the language processing model. As a consequence of the internal forward model, the pentagon can act as a motor speech unit that, once activated, predicts auditory consequences that can be checked against the auditory target. If they match, that unit will continue to be activated, resulting in an articulation that will hit the target. If there is a mismatch, a correction signal can be generated to activate the correct motor unit. The predictions are assumed to be generated by an internal model that receives efferences copies of motor commands and integrates them with information about the current state of the system and experience (learning) of the relation between particular motor commands and their sensory consequences ^67^. The resting state pentagonal synchronization clique enhances the preparatory configuration for the auditory-motor integration to efficiently run when single regions are engaged.

The RS functional network undergoes changes when a task is performed. The phonemic fluency task (Fig. 2 c and d) and the verbs generation task (Fig. 2 e and f) returned very similar patterns of synchronization, both showing the clique formed by SMA and BA L as the most synchronized ones [PLV = 0.725 and PLV = 0.764 respectively]. Subsequently, the PLV hierarchy of the cliques changes according to the task executed. The second most synchronized cliques were (PreMA L, PreMA R) for the verbs generation task [PLV = 0.625] and (AG L, AG R) for the phonemic fluency task [PLV = 0.699]. As opposed to the second most synchronized cliques case, an inversion is shown, being (AG L, AG R) the third most synchronized clique for the verbs generation task [PLV = 0.624] and (PreMA L, PreMA R) for the phonemic fluency task [PLV = 0.595]. The second less synchronized clique was [WA L, WA R, BA R) both for the verb generation [PLV = 0.560] and phonemic fluency [PLV = 0.570] tasks. Finally, for both tasks, the clique (SMG L, SMG R) was the less synchronized one [PLV = 0.538 and PLV = 0.550 for verb generation and phonemic fluency tasks, respectively].

Covert phonemic fluency tasks require phonologic access, verbal working memory, and lexical search activity, which grant a strong activation and lateralization of frontal areas ^68,69^. Sentence completion, such as verb generation, tasks require word recognition and comprehension, understanding of syntactic–semantic relationships between words, planning of a sentence structure and word retrieval ^68^. This leads to increased recruitment of temporoparietal language-related areas, including WA, SMG, and AG ^68,70^.

### 11 Definition of automorphisms and group symmetries

Basic graph theoretical definitions ^47^:

#### Definition 6 (Graph).

*A* graph $G=\left(N_{G},E_{G}\right)$ *is defined by a set* $N_{G}$ *of nodes and a set* $E_{G}$ *of arcs, endowed with two functions* $s,t:E_{G}\to N_{G}$ *that associate a source and target node with each edge.*

#### Definition 7 (Permutation of a graph).

*A* permutation π *of a graph* $G\left(N_{G},E_{G}\right)$ *is a bijective map from the set of nodes* $N_{G}=\{1,\ldots ,N\}$ *to itself*, $\pi \;:N_{G}\to N_{G}$*, that represents the permutation of the node labels.*

For example, the permutation π in the graph of the dual stream baseline connectome in Fig. 1a that maps ${BA}_{L}$ to ${BA}_{R}$ while leaving all the other nodes alone is denoted in cycle notation:

(5) $$\pi =\left({BA}_{L}\;{BA}_{R}\right),$$

meaning that ${BA}_{L}\to {BA}_{R}\to {BA}_{L}$.

#### Definition 8 (Permutation matrix).

A permutation matrix P is an N×N matrix that is obtained from the identity matrix by permuting both rows and columns according to π.

For a graph with N nodes, there are N! permutations, some of which are permutation symmetries or automorphisms, and the rest are not. The set of all permutations of the nodes of a graph $G\left(N_{G},E_{G}\right)$ forms a group $S_{N}=\left\{P_{1},\ldots P_{K}\right\}$ where K=N!. It is called the *symmetric group* (not to be confused with a symmetry group). This set forms a group because the permutations satisfy the associative property, composition (composing two permutations leads to another permutation), and have an identity and inverse.

Basic group theoretical definitions ^71^:

#### Definition 9 (Graph homomorphism).

*A* graph homomorphism φ:G→H (*from a graph* G *to a graph* H) *is a pair of functions* $\varphi_{N}\;:N_{G}\to N_{H}\;and\;\varphi_{E}\;:E_{G}\to E_{H}$ *such that* $s\left(\varphi_{E}(a)\right)=\varphi_{N}(s(a))$ *and* $t\left(\varphi_{E}(a)\right)=\varphi_{N}(t(a))$ *for every edge* $a\in E_{G}$.

#### Definition 10 (Graph isomorphism).

*A* graph isomorphism φ:G→H *is a graph homomorphism whose components* $\varphi_{N}$ and $\varphi_{E}$ *are both bijections.*

#### Definition 11 (Graph Automorphism).

*A* (graph) automorphism (*also called a symmetry permutation or group symmetry*) π:G→G *is an isomorphism from a graph to itself*.

Alternatively, an automorphism is a permutation π:G→G of the vertex set $E_{G}$, such that the pair of vertices i and j forms an edge (i,j) *if and only if* (π(i),π(j)) *also forms a edge.*

That is, an automorphism preserves adjacency and non-adjacency of all the nodes in the graph, and therefore, it is a global symmetry: two edges are adjacent after the permutation if and only if they were adjacent before the permutation.

Any permutation matrix of a permutation π transforms the adjacency matrix into another $A^{\prime }$ as $A^{\prime }=PAP^{-1}$. If P represents an automorphism, then $A^{\prime }=A$, and

(6) $$A=PAP^{-1}.$$

Since the group consists of matrices, we can state this condition differently. Equation (6) holds if and only if the matrix P commutes with A, so PA=AP. Equivalently, the commutator is zero:

(7) $$\left[P,A\right]=PA-AP=0.$$

#### Definition 12 (Symmetry group).

*Graph automorphisms form a group with respect to function composition; this group is denoted by* Aut(G):

(8) $$Aut\left(G\right)=\left\{\pi |\pi \;\text{is}\;\text{a}\;\text{symmetry}\;\text{permutation}\;\text{of}\;G\right\}.$$

The group Aut(G) acts on the set $N_{G}$, mapping the pair $(\pi ,x)\in Aut(G)\times N_{G}$ to $\pi (x)\in N_{G}$. The order of a finite group is the number of its elements. The generators of the symmetry group are a subset of the group set such that every element of the group can be expressed as a composition of finitely many elements of the subset and their inverses.

The set of graph automorphisms permutes certain subsets of nodes among each other. When the symmetry group Aut(G) acts on the network, a given node i is moved by the permutations of the group to various other nodes j. In the language of group theory, the set of all nodes to which i can be moved defines the *orbit* of node i, which in turn defines the orbital partition of the network.

#### Definition 13 (Orbit of a node).

*The orbit of a node* $i\in N_{G}\;for\;the\;symmetry\;group\;Aut(G)$ *is*:

(9) $$𝒮\left(i\right)=\left\{j\in N_{G}|\exists \pi \in Aut\left(G\right)\;s.t.\;\pi \left(i\right)=j\right\}.$$

It can easily be proved that two orbits 𝒮(i) and 𝒮(j) are equal or disjoint, and the union of all orbits equals $N_{G}$. Therefore, the set of all orbits induces a partition of the nodes into mutually disjoint clusters. This set of all orbits forms the *orbital partition*. The same definition can be applied to subgroups H of Aut(G) to obtain H-orbital partitions. When H=Aut(G), we obtain the partition into the fewest subsets.

The orbital partition of the symmetry group corresponds to clusters of nodes that synchronize under a suitable dynamical system of equations that is admissible to the graph. In other words, the orbits guarantee that the cluster synchronization subspace is flow-invariant ^3,5^.

The orbits of the symmetry group (i.e., the partition of $N_{G}$ into orbital equivalence classes, where x is equivalent to y if and only if π(x)=y for some automorphism π) define the automorphism symmetry of G.

### 12 Definition of fibration symmetries

#### Definition 14 (Graph Fibration).

*A* homomorphism ϕ:G→B *is a* fibration *if and only if for every* $a\in E_{B}$ *and every* $x\in N_{G}$ *such that* $\phi (x)=t(a),\;there\;exists\;exactly\;one\;a^{\prime }\in E_{G}$ *such that* $t\left(a^{\prime }\right)=x$ *and* $\phi \left(a^{\prime }\right)=a$. This unique arc $a^{\prime }$ *is called the* lifting of a at x ^7^.

#### Definition 15 (Fibers of the Fibration).

*The* fibers of ϕ *are the sets of nodes of* G *that are mapped to the same node of* B: *these sets form the fiber partition of* $N_{G}$.

#### Definition 16 (Input tree of a node).

*Given a graph* G *and a node* $x\in N_{G}$ *the* input tree of x *in* $G,\;T_{G}(x)$, *is defined recursively as follows: it is a (typically, infinite) rooted tree whose root has as many children as there are in-neighbors of* x *in* G, *and such that the subtrees rooted at each child is the input tree of the corresponding in-neighbor in* G.

It is easy to see that if x, y are two nodes of G that are in the same fiber of *some* fibration, then $T_{G}(x)$ and $T_{G}(y)$ are isomorphic trees.

#### Definition 17 (Symmetry Fibration).

*For every graph* G, *there exists a (base) graph* B *and a surjective fibration* μ:G→B *such that two nodes are in the same fiber of* μ *if and only if* $T_{G}(x)$ *and* $T_{G}(y)$ *are isomorphic. This surjective fibration is essentially unique* ^7^. *It collects all the symmetries of the graph and produces the (minimal) base with the minimal number of fibers: it is called the* symmetry fibration ^11^, *and its fibers define the fibration symmetry of* G.

#### Definition 18 (Cluster synchronization (CS) in a fiber).

Cluster synchronization *occurs for all nodes in a fiber, and they have the same dynamic state as node i, i.e.*,

(10) $$x_{i}\left(t\right)=x_{j}\left(t\right)\;if\;j\in ℱiber\left(i\right),$$

Such a cluster is nontrivial only for fibers of size > 1.

#### Definition 19 (Minimal base of the symmetry fibration).

*Collapsing the nodes in each fiber of the minimal fiber partition to form a single node and respecting the lifting property provides the* minimal base of the symmetry fibration.

### 13 Definition of the dual (dorsal) stream baseline connectome of language

According to the dual stream model introduced in Methods Sec. 7, it is known that human language relies on two primary white-matter pathways: the dorsal stream, which is related to sensorimotor integration, and the ventral stream, which is related to speech comprehension ^51^. The tracts we are interested in are the primary tracks of the dorsal stream, which is formed by white matter tracks of the superior longitudinal fasciculus (SLF) arcuate fasciculus (AF) system. The AF connects the inferior frontal gyrus (BA) to the posterior superior temporal gyrus (WA); the SLF connects BA and PreMA to the inferior parietal areas (SMG and AG) ^51,52^. The frontal aslant tract (FAT) connects BA with the SMA, serving the verbal fluency components of language ^53^. Sensorimotor integration culminates in the BA and ventral PreMA, which are responsible for articulatory planning ^56^. Two parallel dorsal pathways have also been described ^17^. One connects the PreMA (dorsal pathway I) and BA (dorsal pathway II) to the WA, with the first predominantly supporting sound-to-motor mapping and the second supporting higher-level language processes. It is also known that PreMA represents a crucial speech production hub thanks to its coupling with the SLF. Preservation of this cortical-subcortical connection is crucial for speech integrity and represents an anatomical constraint to cortical plasticity ^72^. Additionally, homologous right- and left-sided cortical areas are likely connected by the corpus callosum, the main inter-hemispheric commissure, which enables communication between the two cerebral hemispheres ^73^.

These tracks constitute the primary dual-stream baseline connectome: the set of routes composing the information transfer highway within the language network. They are displayed in Fig. 1a and show a highly symmetric structure since the automorphisms are the same as the symmetry fibrations as seen in Fig. 4a.

### 14 Analysis of symmetries of the dorsal stream baseline connectome

**Figure 7:**
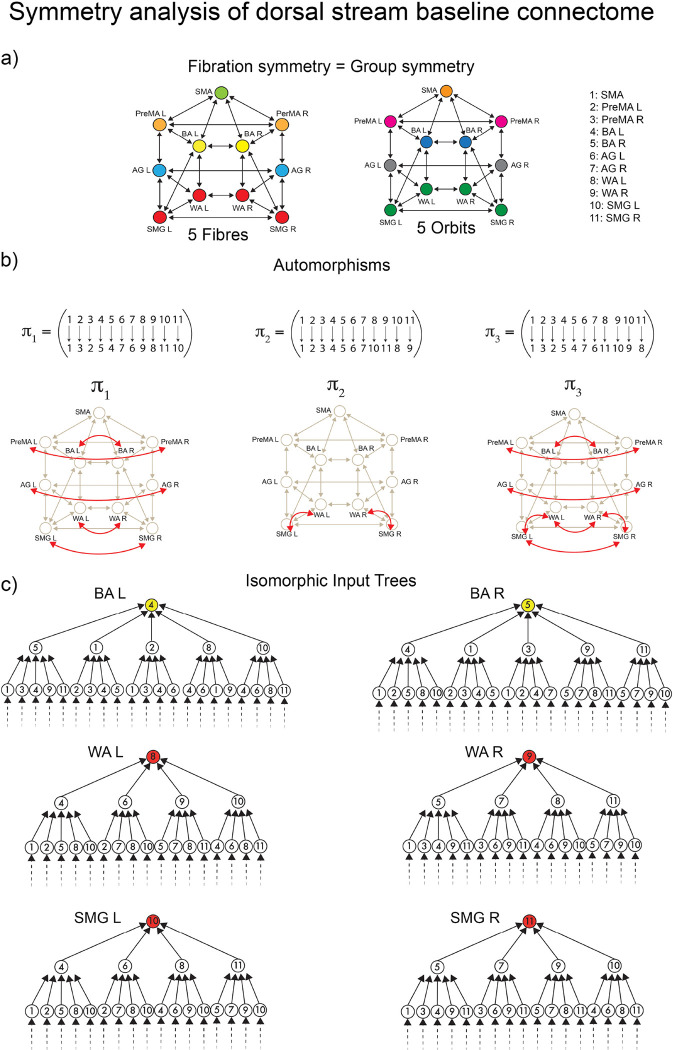
Extended Data Fig. 7. Group and fibration symmetry analysis of the dual stream (dorsal) baseline connectome of language. **(a)** Orbital and fiber partition of this connection is the same, indicating a high level of symmetry of the ‘highway’ network. **(b)** Two generators of the symmetry group of the baseline connectome. Left: the left-right (mirror) global symmetry $\pi_{1}$. Center: the symmetry permutation $\pi_{2}=\left({WFA}_{L}{SMG}_{L}\right)\left({WA}_{R}{SMG}_{R}\right)$. Applying these two symmetries to each node in the graph generates the orbits. Right: composition between $\pi_{3}=\pi_{1}\cdot \pi_{2}$. **(c)** Example of two sets of isomorphic input trees giving rise to the main fiber made of four ROIs WA and SMG left and right, and one sample of the bilateral fiber BA left and right (the remaining bilateral fibers are similar). This graph has the same group and fibration symmetry.

We perform a full symmetry analysis (including group and fibration symmetry) of the dorsal stream baseline connectome in Fig. 7.

McKay’s Nauty algorithm ^50^ is used to calculate the automorphisms of the connectome. The connectome contains 11! = 39,916,800 possible permutations of its 11 ROIs. From this, only a few are permutation symmetries. There are eight generators of the symmetry group of this connectome:

(11) $$Aut\left(G\right)=\left\{\pi_{j}|\pi_{j}\;\text{is}\;\text{a}\;\text{symmetry}\;\text{permutation}\;\text{with}\;j=0,\;2\right\},$$

where the automorphisms (including the identity) in cycle notation are (Fig. 7b):

$$\pi_{0}=Id$$

$$\pi_{1}=\left({PreMA}_{L}{PreMA}_{R}\right)\left({BA}_{L}{BA}_{R}\right)\left({AG}_{L}{AG}_{R}\right)\;\;\left({WA}_{L}{WA}_{R}\right)\left({SMG}_{L}{SMG}_{R}\right)$$

$$\pi_{2}=\left({WA}_{L}{SMG}_{L}\right)\left({WA}_{R}{SMG}_{R}\right)$$

The actions of this symmetry group generate five orbits, which is the orbital color partition that we see in Fig. 4a and in Fig. 7a:

$${𝒮}_{1}=\left\{{PreMA}_{L},{PreMA}_{R}\right\}$$

$${𝒮}_{2}=\left\{{BA}_{L},{BA}_{R}\right\}$$

$${𝒮}_{3}=\left\{{AG}_{L},{AG}_{R}\right\}$$

$${𝒮}_{4}=\left\{{WA}_{L},{WA}_{R},{SMG}_{L}{SMG}_{R}\right\}$$

$${𝒮}_{5}=\{SMA\}$$

The fibration analysis is done by searching for the minimal balanced coloring of the network using the refinement algorithm of Kamei and Cock ^49^ employed by Morone *et al.* in ^11^. The minimal balanced coloring of the graph corresponds to a balanced coloring with a minimal number of colors. Each cluster of balanced coloring is a fiber. The resulting coloring is shown in Fig. 7a, left. It shows the existence of five fibers; each fiber is also an orbit. The minimal partition obtained by the fibers is the same as the minimal orbital partition. This implies that the group symmetry of this graph is the same as the fibration symmetry of the graph.

The fibers are:

$${ℱiber}_{1}=\left\{{\text{PreMA}}_{L},{\text{PreMA}}_{R}\right\}$$

$${ℱiber}_{2}=\left\{{BA}_{L},{BA}_{R}\right\}$$

$${ℱiber}_{3}=\left\{{AG}_{L},{AG}_{R}\right\}$$

$${ℱiber}_{4}=\left\{{WA}_{L},{WA}_{R},{SMG}_{L}{SMG}_{R}\right\}$$

$${ℱiber}_{5}=\{SMA\}$$

Generally, the orbital partition obtained from automorphisms does not necessarily need to coincide with the balanced coloring obtained from the fibration analysis. Fibers capture more symmetries than orbits. Thus, the number of fibers is always equal to or smaller than the number of orbits. Moreover, an orbit is always part of a fiber, but a fiber may not be part of an orbit. When these two partitions are the same, the two symmetries are the same, too, implying a high symmetry state.

The analysis of the input trees of this connectome is shown in Fig. 7c for the main fiber of 4 ROIs, ${ℱiber}_{4}$ and a representative bilateral fiber ${ℱiber}_{2}$. This shows the isomorphism between the input trees of ROIs within a fiber. This analysis complements the fibration analysis of balanced coloring. The same analysis is done below for the RS and task-based inferred networks.

### 15 Integer linear program for symmetry-driven inference of the structural network to satisfy cluster synchronization

The way the functioning of the brain is connected to its underlying structure adjusts according to the requirements of the brain activity ^13,14^. Thus, the same baseline highway can give rise to different functional states, e.g., RS or language task of verbal and fluency, given by different synchronized coloring patterns (e.g., Fig. 2a, c, e, respectively). Consistent with this idea, a given functional network (at rest or task) is sustained by a specific configuration of the connectome, in a way, strictly depends on the activity itself. A condition for such matching to exist is that a modification of the connectome displayed in Fig. 1a is introduced. Given the baseline connectome, which represents the communication highway, only a subset of routes are needed to guarantee the existence of the synchronous clusters (fibers/orbits) of the functional network.

The crucial step of this scheme to infer the structural network from the functional network is the fibration/group symmetry partitioning and the iterative decimation process for the coloring matching. The partition problem is weakly NP-Hard ^74^, but it has been shown that solving the directed/undirected in-balanced K-coloring problem solves the partition problem ^33^. In particular, the problem of finding removals that satisfy the coloring condition can be formulated as a mixed integer program that is solvable for modestly sized instances. We follow a similar approach in this paper and formulate the problem of finding the minimum perturbations to induce a minimal balanced coloring as an integer linear program, i.e., an optimization problem where the decision variables are all integer. The objective and constraint functions are linear. We then solve the integer linear programs with the solver Gurobi ^75^.

We consider a directed graph, G=(V,E), where V denotes the set of nodes, and E denotes the set of directed edges (an undirected edge is considered as two directed edges). Also, we denote n=|V| and m=|E| as the number of nodes and directed edges, respectively. We also define

(12) $$E^{C}=\{ij\;:i,j\in V,ij\notin E\}$$

as the set of ordered pairs of nodes for which no directed edge exists in G, which we refer to as *non-edges*. These ordered pairs represent potential edges that could be added to the graph G. We let 𝒮 denote a coloring of G, i.e., 𝒮 is the collection of sets partitioning V. This coloring, 𝒮, is provided by the CS from fMRI synchronization in different engagements of the brain function, Fig. 2a, c, e. We define α, β as constant parameters that govern the objective’s relative importance between edge removal and edge addition. *We wish to determine the minimum number of edges to add or remove so that* S *is a balanced coloring of* G, *i.e*., 𝒮 *satisfies Definition 5 for* G. Our integer programs are guaranteed to find a balanced coloring but are not guaranteed to find a minimal balanced coloring. However, in our experiments, a minimal balanced coloring was found in all cases we tested.

The model’s three families of binary decision variables are defined as follows. For ij∈E,

(13) $$r_{ij}=1\;\text{if}\;\text{edge}\;ij\;\text{is}\;\text{removed,}\;0\;\text{otherwise.}$$

For $ij\in E^{C}$,

(14) $$a_{ij}=1\;\text{if}\;\text{non-edge}\;ij\;\text{is}\;\text{added,}\;0\;\text{otherwise.}$$

For P,Q,R∈𝒮 with P≠Q and for i∈P, j∈Q

(15) $$s_{ijR}=1\;\text{if}\;i\;\text{and}\;j\;\text{are}\;\text{imbalanced}\;\text{on}\;R$$

and 0 otherwise. The role of the linear constraints below are to set up a set of linear equalities and inequalities that, if satisfied by these decision variables, cause the resulting perturbed graph to be a minimal balanced coloring.

The objective function is to minimize the weighted sum of edges removed and edges added. The function is then defined as:

(16) $$f_{\alpha ,\beta }\left(r,a\right)=\alpha \sum_{ij\in E}r_{ij}+\beta \sum_{ij\in E^{C}}a_{ij}.$$

The main constraint assures that 𝒮 is a balanced coloring of the perturbed graph G.


(17) $$\begin{array}{l} \sum_{ip\in E:i\in S}\left(1-r_{ip}\right)+\sum_{ip\in E^{C}:i\in S}a_{ip}=\\ \sum_{iq\in E:i\in S}\left(1-r_{iq}\right)+\sum_{iq\in E^{C}:i\in S}a_{iq};p,q\in T;S,T\in 𝒮. \end{array}$$

Constraints (17) exist for every pair of nodes p, q that are the same color and for every color set. Note that for a given edge ij∈E, the quantity $1-r_{ij}$ is 1 if the edge is not removed and 0 if it is removed. Also, for $ij\in E^{C}$, the quantity $a_{ij}$ is 1 if ij is a newly created edge and 0 otherwise. Thus, the left-hand side of (17) represents the edges that enter into a given node p from the color set S, and the right-hand side represents the edges entering node q from the color set S. Using the same sums, (18) ensure that the in-degree is at least one for every node:

(18) $$\sum_{ip\in E}\left(1-r_{ip}\right)+\sum_{ip}a_{ip}\geq 1,\;p\in V.$$

The following constraints are valid for minimal balanced colorings, i.e., they are necessary but not sufficient.


(19) $$\begin{array}{l} \sum_{ip\in E:i\in R}\left(1-r_{ip}\right)+\sum_{ip:i\in R}a_{ip}-\\ \left(\sum_{iq\in E:i\in R}\left(1-r_{iq}\right)+\sum_{iq:i\in R}a_{iq}\right)\geq s_{pqR}-ns_{qpR};\\ p\in S;\;q\in T;R,S\neq T\in 𝒮, \end{array}$$

(20) $$\begin{array}{l} \sum_{iq\in E:i\in R}\left(1-r_{iq}\right)+\sum_{iq:i\in R}a_{iq}-\\ \left(\sum_{ip\in E:i\in R}\left(1-r_{ip}\right)+\sum_{ip:i\in R}a_{ip}\right)\geq s_{qpR}-ns_{pqR};\\ p\in S;\;q\in T;R,S\neq T\in 𝒮, \end{array}$$

(21) $$\begin{array}{l} \;s_{pqR}+s_{qpR}\leq 1,\\ p\in S;q\in T;R,S\neq T\in 𝒮, \end{array}$$

(22) $$s_{pqR}+s_{qpR}\leq 1;$$

(23) p∈S;q∈T;S,T∈𝒮

The inequalities (21) keep at most one of the two binary variables $s_{pqR}$ to be equal to one for every color R. If both are zero, then the inequalities (19) and (20) would force p and q to be balanced for the color R. If one is zero, the total in-adjacent nodes of color R would be at least one different for p and q. In particular, for color R, if $s_{pqR}=1$ and $s_{qpR}=0$, then the number of in-adjacent nodes to p is at least one greater than that to color q. The converse is also true. The inequalities (23) force that one of $s_{pqR}$ or $s_{qpR}=0$ is equal to one for at least one color R. This is a necessary but not sufficient condition for the coloring to be *minimal*. For example, if two color partitions have no edges between them, the same number of edges to all other colors, and the same positive number of internal edges, then (23) is satisfied as their different colors will register as an imbalance. However, the union of these two color partitions is balanced and has one less color, i.e., the coloring is no longer minimal. That being said, our experiments yielded strong evidence that the necessary condition sufficiently enforces the minimal balanced condition in practice, as we found a minimal balanced coloring for all of our test cases.

The complete model is then:

(24) $$\begin{array}{ll} minf_{\alpha ,\beta }(r,a) & \\ \text{subject}\;\text{to} & \left(17\right),\left(18\right),\left(20\right),\left(20\right),\left(21\right),\left(23\right),\\ & r_{ij},a_{k\ell },s_{pqR}\in \left\{0,1\right\},ij\in E,\\ & k\ell \in E^{C},p\in P,q\in Q,P\neq Q,R\in 𝒮. \end{array}$$

where Eq. (23) within equation above is a reference to select only one of its sub-equations.

The uniqueness of the solution is tested by developing an independent solver based on the quasi-fibration framework developed in ^34^. In all cases considered, we find the same solution using the quasi-fibration formalism and MILP.

Due to its large complexity, the brain can never have exact symmetries, even within a single connectome. Structural brains are all different, but a certain level of ideal symmetry must be common to all of them to guarantee the performance of an average synchronization pattern, despite not all structural brains being identical. We apply the inference algorithms to those group-average synchronization networks and connectomes at the mesoscopic level, as shown in Figs. 2 and 4 which should be interpreted as idealized networks.

### 16 Symmetry breaking in physics and the brain

Most symmetry laws in physics are broken in one way or another. One such mechanism is spontaneous symmetry breaking, where the laws of physics remain symmetric, but the system’s ground state exhibits a lower symmetry than the full system, as in a paramagnetic-to-ferromagnetic phase transition ^37^. For temperatures below the critical value $T_{C}$, the magnetic moments of the atoms of ferromagnetic material are partially aligned within magnetic domains, producing a net magnetic moment even though the atoms interact through a spin-spin interaction, which is invariant under rotation. Thus, the rotational invariant symmetry of the system is broken into this ground state with a non-zero magnetic moment. As the temperature increases, this alignment is destroyed by thermal fluctuations and the net magnetization progressively reduces until vanishing at $T_{C}$. The orientation of the magnetization is random. Each possible direction is equally likely to occur, but only one is chosen at random, resulting in a zero net magnetic moment. So, the rotational symmetry of the ferromagnet is manifest for $T>T_{C}$ with zero magnetic moment, but is broken by the arbitrary selection of a particular (less-symmetrical) ground state with non-zero magnetic moment for $T<T_{C}$.

Another type is explicit symmetry breaking, where the dynamics are only approximately symmetric, yet the deviation caused by the breaking forces is minimal. Hence, one can consider the symmetry violation as a small correction in the system. An example is the spectral line splitting in the Zeeman effect due to a magnetic interaction perturbation in the Hamiltonian of the atoms involved.

In the present work, we implemented a symmetry-driven algorithm based on a mixed integer linear program to infer the structural network associated with each balanced coloring of the functional network obtained experimentally in different tasks. By applying this novel framework to healthy subjects performing standard language tasks, we obtained a functional language anatomy which is consistent with the common understanding of speech processing.

The symmetry-breaking we find in the brain is manifested in the following:

1. The evidence of an underlying highly symmetrical connectome between the language areas (group symmetry = fibration symmetry) with a novel central fiber made of 4 ROIs.
2. The evidence of a symmetrical language network representation during resting state as a consequence of the overall synchronization dynamics with a novel pentagonal fiber at the core of the network made of SMA, PreMA and WA. This network presents only fibration symmetries but no group symmetries thus, the resting state engagement breaks the global group symmetries of the baseline connectome. That is, even though the ROIs are synchronized in pairs with left-right symmetry, the underlying structural network does not have the global left-right symmetry. In fact, it has no automorphisms at all. This is remarkable. The only surviving symmetry is the local fibration.
3. The characterization of the transition between resting state and language tasks as further broken symmetry, but this time of the fibration symmetry (the group symmetry remains broken). The evidence of a breaking of symmetry resulting in two novel central fibers (BA L-SMA) and (WA L-WA R-BR R).
4. The evidence of slightly different engagement of the comprehension center formed by frontotemporal-parietal language areas in the phonemic fluency and verb generation tasks supported by the same pattern of communication routes, i.e., the same structural connectivity. Thus, while the communication routes are the same for the two tasks, frontal and parietal regions are characterized by different levels of bilateral synchronization (different rearrangement to communication) according to the task executed: frontal area is more synchronized during verb generation, and parietal areas are more synchronized during noun generation.
5. Possible applications will include the analysis of broken symmetries in neurological disorders and correlation with patients’ clinical performance. Some neurological conditions compromise the synchronization in the brain (tumors, stroke, any focal lesion), affecting its coherent activity. By applying our method to these patients, we could shed light on biomarkers that could predict symptoms and patients’ prognosis.

### 17 Analysis of symmetries of the RS structural network

**Figure 8:**
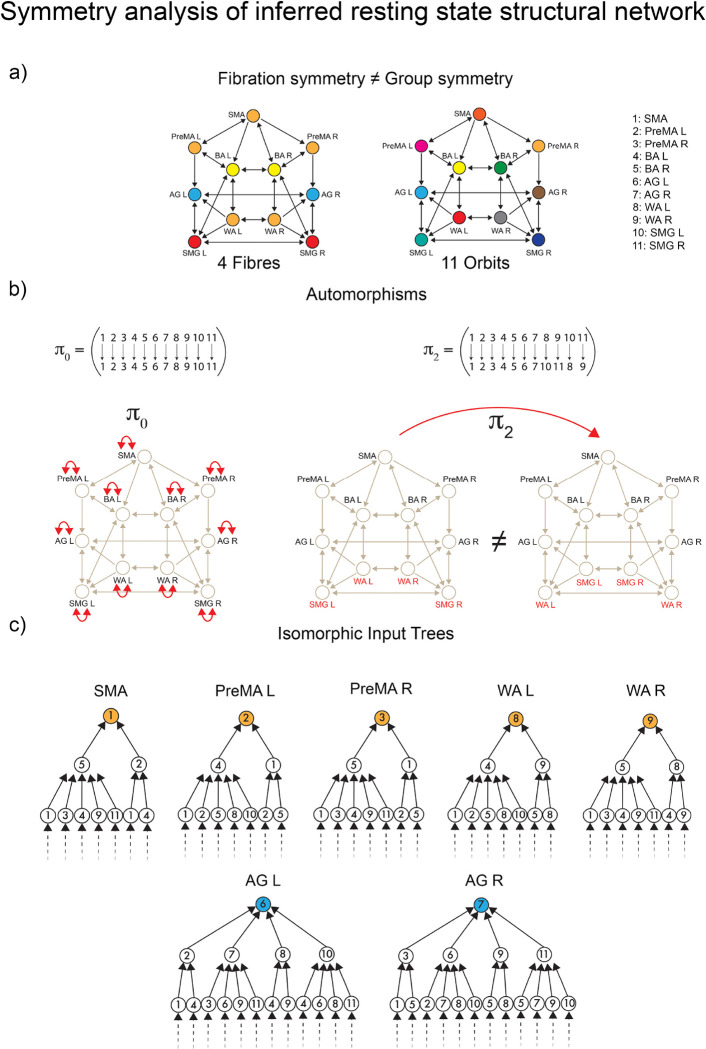
Extended Data Fig. 8. Group and fibration symmetry analysis of the inferred structural network supporting the resting state. **(a)** Orbital and fiber partition of the structural network. This network has no automorphisms, except for the trivial identity leading to a trivial orbital partition of 11 colors where each ROI is its own (trivial) orbit. This implies that the symmetry group of the underlying baseline connectome of Fig. 7a has been completely broken in the resting state. However, the fibration symmetry remains. Fibration analysis reveals four fibers as observed in the four balanced colorings of the network. **(b)** There are no (non-trivial) automorphisms in this network. Only the identity $\pi_{0}$ is a trivially global symmetry (left). The permutation $\pi_{2}$ showing in the right is not a symmetry. Yet, WA left and right are still locally symmetric under a fibration. **(c)** Example of isomorphic input trees of the ROIs in the main fiber made of the pentagonal fiber: PreMA left and right, SMA and WA left and right, and one sample of the bilateral fiber AG left and right (the remaining bilateral fibers are similar).

We perform a full symmetry analysis (group and fibration symmetry) of the inferred resting state structural network in Fig. 8.

First, McKay’s Nauty algorithm ^50^ is used to calculate the automorphisms of the network. Out of the 11! = 39,916,800 possible permutations of its 11 ROIs only the identity $\left[\pi_{0}=Id\right]$ is an automorphism (Fig. 8b, left). Any other permutation is not a symmetry. For instance, if we implement the permutation

(25) $$\pi_{2}=\left({WA}_{L}{SMG}_{L}\right)\left({WA}_{R}{SMG}_{R}\right),$$

we obtain a different graph (Fig. 8b, right).

Accordingly, the resting state structural network has only trivial group symmetry. Colloquially, we say that this network has no group symmetry. Since the only automorphism is the trivial identity, each node has its orbit. Thus, there are eleven orbits: one for each node, Fig. 8a, right. If compared to the baseline connectome that shows five orbits, Fig. 7a right, this represents a group symmetry breaking.

The fibration symmetry analysis is done by finding the balanced coloring (fibers) using the refinement algorithm of Kamei and Cock ^49^ and Morone *et al.* ^11^. Figure 8a left shows the resulting minimal balanced coloring. When compared to the baseline connectome Fig. 7a left, an increase of fibration symmetry is obtained since now we observed a smaller number of fibers. Recall that the most symmetric graph is that with a single color, and the least symmetric graph is the one with N colors for a graph with N nodes. While in the baseline, we have five fibers, in the resting state the number of fibers is four.

When compared to the orbital partition of the same graph, Fig. 8a, right, we find that this graph has fibration symmetry but no group symmetry. The global symmetry has been fully broken by engaging the brain in RS, but the local symmetry remains, and it is enhanced in RS in comparison to the original symmetry of the connectome.

The fibers are:

$${ℱiber}_{1}=\left\{SMA,{\text{PreMA}}_{L},{\;\text{PreMA}}_{R},{WA}_{L},{WA}_{R}\right\}$$

$${ℱiber}_{2}=\left\{{BA}_{L},{BA}_{R}\right\}$$

$${ℱiber}_{3}=\left\{{AG}_{L},{AG}_{R}\right\}$$

$${ℱiber}_{4}=\left\{{SMG}_{L}{SMG}_{R}\right\}$$

The analysis of the input trees is shown in Fig. 8c for the main fiber of 5 ROIs, ${ℱiber}_{1}$ and a representative bilateral fiber ${ℱiber}_{3}$. This complements the fibration analysis of this graph.

### 18 Analysis of symmetries of the task structural network

**Figure 9:**
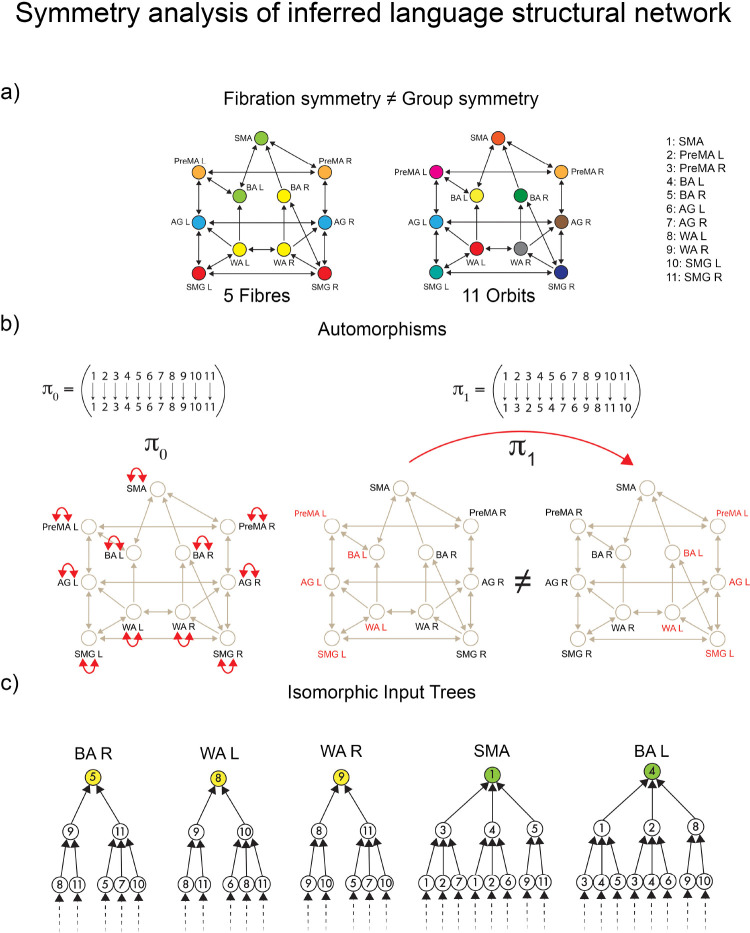
Extended Data Fig. 9. Group and fibration symmetry analysis of the inferred structural network supporting the language task. **(a)** Orbital and fiber partition of the structural network. This network has no automorphisms, except for the identity leading to a trivial orbital partition of 11 colors where each ROI is its own (trivial) orbit. The symmetry group of the resting state network of Fig. 8a remains fully broken in the task. The local fibration symmetry is broken from the resting state due to the lateralization imposed by the task. BA left, and right are broken, and they are recruited by the SMA and WA, respectively, belonging now to two different fibers (colors). The number of fibers is now five, implying a broken fibration symmetry from the resting state since there are more fibers (less symmetry) in the task. **(b)** Like in the resting state, this network has no (non-trivial) automorphisms. For instance, the global left-right group symmetry is broken, as the figure indicates. **(c)** Example of isomorphic input trees of the ROIs in the largest fiber made of the three ROIs: WA left and right, and BA right, and the fiber formed by SMA and BA left.

We perform a full symmetry analysis (including group and fibration symmetry) of the inferred task structural network in Fig. 9.

Similar to the RS network, McKay’s Nauty algorithm ^50^ shows that this graph has no automorphisms except for the trivial identity. From the 11! allowed permutations, only the identity $\left[\pi_{0}=Id\right]$ is a symmetry (Fig. 9b, left). For instance, if we implement the permutation

(26) $$\pi_{1}=\left({PreMA}_{L}{PreMA}_{R}\right)\left({BA}_{L}{BA}_{R}\right)\left({AG}_{L}{AG}_{R}\right)\left({WA}_{L}{WA}_{R}\right)\left({SMG}_{L}{SMG}_{R}\right)$$

we obtain a different graph (Fig. 9b, right), exactly as we found for in RS (Fig. 8b).

Since there are no (non-trivial) automorphisms, we obtain eleven orbits (one for each node, Fig. 9a, right) for the task network as in RS. Thus, the baseline connectome’s global symmetry remains broken in the language task.

The fibration symmetry analysis for this connectome gives rise to a the balanced coloring partition seen in Fig. 9a, left. We found five fibers:

$${ℱiber}_{1}=\left\{\;\text{SMA,}\;{BA}_{L}\right\}$$

$${ℱiber}_{2}=\left\{{PreMA}_{L},{\text{PreMA}}_{R}\right\}$$

$${ℱiber}_{3}=\left\{{AG}_{L},{AG}_{R}\right\}$$

$${ℱiber}_{4}=\left\{{BA}_{R},{WA}_{L}{WA}_{R}\right\}$$

$${ℱiber}_{5}=\left\{{SMG}_{L}{SMG}_{R}\right\}$$

If compared to the resting state connectome Fig. 8a, a decrease of fibration symmetry is obtained. In RS, we have four fibers, and in the task, we have five. This represents a fibration symmetry breaking (more fibers means less fibration symmetry). This local symmetry breaking is the product of the breaking of left-right local symmetry in the Broca area due to language lateralization. Recall that the left-right global symmetry has been fully broken in the RS state and remains broken here. This local breaking of symmetry is done by the synchronization of Broca left with SMA, and the independent synchronization of Broca right to Wernicke left and right. These two areas remain locally left-right symmetric. This produces the main two fibers controlling the language network ${ℱiber}_{1}$ and ${ℱiber}_{4}$. The analysis of the input trees of these fibers is shown in Fig. 9c.

## Figures and Tables

**Figure 5:**
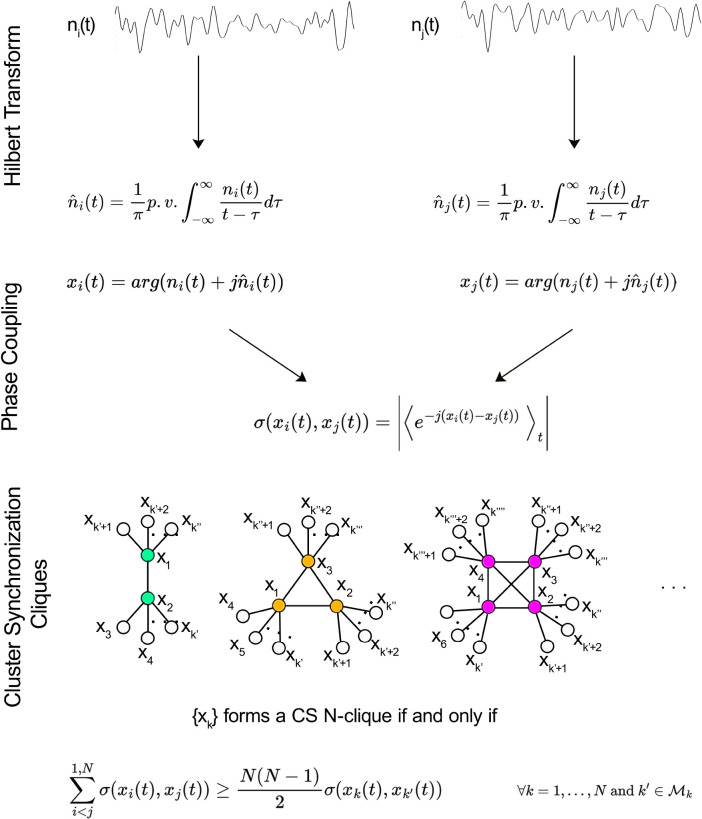
Extended Data Fig. 5. Schematics of the synchronization clustering algorithm. Pairs of time series coming from pairs of cerebral ROIs are Hilbert transformed and entered in the phase-locking value calculation. Once all the pairs of regions of interest are included in the calculation, the synchronization clustering algorithm is implemented.

**Figure 6:**
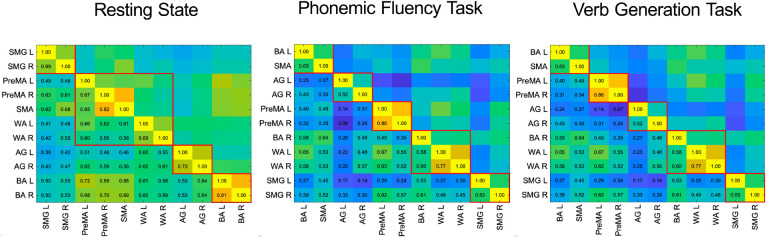
Extended Data Fig. 6. Single subject phase locking value matrices. **(a)** Phase locking value matrix for the resting state condition for a typical subject. **(b)** Phase locking value matrix for the phonemic fluency task condition. **(c)** Phase locking value matrix for the verb generation task condition. The red-lined boxes are visual indicators for the CS, and clusters are shown according to the order used for the average matrices shown in Fig. 2.
